# Effective nationwide school-based participatory extramural program on adolescent body mass index, health knowledge and behaviors

**DOI:** 10.1186/s12887-017-0975-9

**Published:** 2018-01-16

**Authors:** Moonseong Heo, Camille C. Jimenez, Jean Lim, Carmen R. Isasi, Arthur E. Blank, David W. Lounsbury, Lynn Fredericks, Michelle Bouchard, Myles S. Faith, Judith Wylie

**Affiliations:** 1 Department of Epidemiology and Population Health, Albert Einstein College of Medicine, 1300 Morris Park Avenue, Bronx, NY 10461 USA; 2 HealthCorps, 33 Irving Pl, 3rd Floor, New York, NY 10003 USA; 3 Department of Family and Social Medicine, Department of Epidemiology and Population Health, Albert Einstein College of Medicine, Harold and Muriel Block Building, Room 409, 1300 Morris Park Avenue, Belfer 13-th floor, Bronx, NY 10461 USA; 4 FamilyCook Productions, 330 East 43rd street, Ste. 704, New York, NY 10017 USA; 5 Department of Counseling, School, and Educational Psychology, Graduate School of Education, 420 Baldy Hall, University of Buffalo – SUNY, Buffalo, NY 14260-1000 USA

**Keywords:** Adolescent obesity, Health behavior, High school, HealthCorps

## Abstract

**Background:**

Adolescent obesity is a major public health concern. Open to all high school students regardless of weight status, HealthCorps is a nationwide program offering a comprehensive high school-based participatory educational program to indirectly address obesity. We tested a hypothesis that the HealthCorps program would decrease BMI z-scores among overweight or obese students, and reduce obesity rates, and evaluated its effects on health knowledge and behaviors.

**Methods:**

HealthCorps aimed to improve student knowledge and behaviors regarding nutrition quality, physical activity, sleep, breakfast intake, and mental resilience. Participating students received through HealthCorps coordinators weekly or bi-weekly classroom lessons either for a semester or a year in addition to various during- and after-school health-promoting activities and mentorship. Self-reported height and weight were collected along with questionnaires assessing knowledge and behaviors during 2013-2014 academic year among 14 HealthCorps-participating New York City high schools. This quasi experimental two-arm pre-post trial included 611 HealthCorps and 221 comparison arm students for the analytic sample. Sex-specific analyses stratified by weight status were adjusted for age and Hispanic ethnicity with clustering effects of schools and students taken into account.

**Results:**

HealthCorps female overweight/obese and obese student had a significant decrease in BMI z-scores (post-pre delta BMI z-score = −0.16 (95%CI = (−0.26, −0.05), *p* = 0.004 for the former; and = −0.23 (−0.44, −0.03), *p* = 0.028, for the latter) whereas comparison female counterparts did not. The HealthCorps students, but not the comparison students, had a significant increase for all knowledge domains except for the breakfast realm, and reported a greater number of significant behavior changes including fruit and vegetable intake and physical activities.

**Conclusions:**

The HealthCorps program was associated with reduced BMI z-score in overweight/obese and obese female adolescents, with enhanced health knowledge and behavior for both sexes. With its wide reach, this may be a promising program to help combat adolescent obesity in schools.

**Trial registration:**

This study is registered as a clinical trial at the ClinicalTrials.gov registry with trial number NCT02277496 on September 10, 2014 (Retrospectively registered).

**Electronic supplementary material:**

The online version of this article (10.1186/s12887-017-0975-9) contains supplementary material, which is available to authorized users.

## Background

Adolescent obesity is a major public health concern that affects health status not only during adolescence but also during adulthood. [1] The effects of adolescent obesity on a range of current and later life diseases or psychological stresses are well documented in literature. [2–8], and include cardiovascular mortality during adulthood. [9] Analysis of the National Health and Nutrition Examination Survey 2013-2014 data indicated that among adolescents aged 12 to 19 years the obesity prevalence increased steadily from 10.5% in 1988-1994 to 20.6% in 2013-2014 although the prevalence has leveled off somewhat in recent years. [10] It is unclear if the leveling off is related to interventions focusing on the Healthy People 2020 objective to reduce prevalence of adolescents with obesity. [11, 12]

Evidence-based obesity interventions in school settings would appear to be a logical strategy for helping to achieve this objective. [13] Several school-based trials for prevention or control of childhood and adolescent obesity have been conducted, and the results have been somewhat inconsistent. [14–17] School-based interventions that target students with obesity could inadvertently stigmatize them, be it through internal self-perception or external recognition by peers or others. [18–20] Nonetheless, to our knowledge, no school-based trial to date has utilized an extramural program to address adolescent weight improvement in a high school setting while also minimizing potential weight stigmatization.

HealthCorps Inc. (www.healthcorps.org), an United States nationwide extramural non-profit non-governmental organization, offers a unique comprehensive high school-based voluntary participatory educational program that is open to all students regardless of their weight status. It is estimated that more than 1.8 million adolescents to date have been exposed to the program since its foundation in 2003 when the HealthCorps program was the first of its kind that was implemented in New York City (NYC) public high schools with intense and challenging urban inner city environments. The program does not directly intervene on weight issues but offers substantive wellness education that could help students achieve a healthier form. Furthermore, the setting of HealthCorps high schools was also unique in terms of demography and inadequate resources to promote health behaviors. The program focuses on improving the health knowledge and behaviors on: nutrition, physical activities, breakfast intake, adequate sleep, and mental resilience.

Our recent program evaluation suggested that the HealthCorps programming during 2012-2013 academic year was generally effective for enhancing knowledge and behaviors among students in NYC public high schools. [21] However, data collection did not include height and weight and thus did not permit assessing the effects of the program on weight improvement. To address this, HealthCorps collected self-reported weight and height data during the 2013-2014 academic year in NYC high schools with HealthCorps programming.

The present study is primarily aimed to test whether the HealthCorps program would improve weight status represented by body mass index (BMI; kg/m^2^) z-score and obesity class. We hypothesized that the program would decrease BMI z-scores among overweight or obese students, and reduce obesity rates. Secondarily, we aimed to identify knowledge and health behavior domains that would be increased with the program.

## Methods

The present analysis was conducted based on students’ HealthCorps survey responses and their self-reported weight and height data from New York City (NYC) high schools before and after participating in HealthCorps programming during the 2013-2014 academic year, although during this year, a total of 62 schools over 13 states participated in HealthCorps program. The present study focused on implementation of the HealthCorps programming in selected NYC high schools. The selection criteria for the NYC HealthCorps schools were as follows: [21] (1) must be a public high school; (2) must have 50% or more students eligible for free or reduced-price meals; and (3) must lack any existing programs aimed at improving nutrition, physical activity and mental resilience behavior. Although a total of 15 schools were selected, survey data were not available from 1 school. Whites were a minority racial group in all of the selected 14 NYC schools with HealthCorps programming.

The study design was a two parallel arm quasi-experimental pre-post comparison design as neither the schools nor the students were randomly assigned to accommodate real-world settings and constraints as much as possible, and students’ participation in the HealthCorps program was voluntary. This study was approved by the Institutional Review Boards of Albert Einstein College of Medicine and NYC Department of Education.

### HealthCorps coordinators

HealthCorps programming consists of a curriculum delivered in classrooms, through mentorship, and through a variety of during- and after-school health-promoting activities (www.healthcorps.org/resources/the-curriculum). All of these activities were coordinated and delivered by intensively trained well-qualified program coordinators each of whom is designated to a single school (https://www.healthcorps.org/become-a-coordinator/). All Coordinators are trained on all HC program components for 3 weeks in the summer as well as a week of professional development in the winter recession. Through weekly check-ins and reports, program supervisors ensure all program components are being implemented at their coordinators’ schools. Each supervisor visits a site at least once a year to observe delivery of the curriculum also to ensure standardization. Depending on school policies or school culture, however, coordinators are encouraged to tailor the program to meet the needs of their specific school and community. As such, the coordinators often work in conjunction with a school wellness council and its members.

### HealthCorps activities

Coordinators teach approximately 10 classroom HealthCorps lessons weekly or bi-weekly and for either a semester or a year. These lesson topics include developing tools that build mental resilience, healthy eating habits, and physical fitness. The coordinators also provide mentoring to students and staff with various health-related resources and action plans that address meeting personal health goals. Coordinators also co-facilitate wellness councils, comprised of staff members and students, who utilize the Alliance for a Healthier Generation platform (www.healthiergeneration.org) and inventory to address gaps in the school’s health policies and programming in order to design action plans to improve nutrition, physical fitness and mental resilience of their students. Weekly afterschool clubs focus on nutrition, physical fitness and/or mental resilience whose contents were determined by the annual HealthCorps Community Needs Assessment instrument that systematically identifies areas of programming need at each school.

Activities outside the classroom included lunchroom “Café-o-Yeas” food samplings, Teen Battle Chef and other cooking programs, Youth Lead Action Research, and annual Highway to Health school and community-wide Festivals. The “Café-o-Yeas” is a monthly activity in that coordinator and students share samples of healthy foods with other students at lunchtime at a booth. The Teen Battle Chef program involves culinary coaching and cooking “battles” to help students learn how to cook healthy meals and build their public speaking, teamwork and leadership skills. [22] Finally, the Youth Lead Action Research program helps students develop research abilities so that they can identify health needs in their school or community using surveys, interviews or photovoice web tools (www.photovoice.org) and then develop specific projects that meet those needs.

Depending on restrictions or schedules of schools, lengths of the program were a semester- or year-long. Regardless of the length, however, the school-level dosage of the program was identical and the aforementioned program activities were applied to all participating schools. Implementation process of the program activities was determined specific to each school depending on its needs and overall culture identified based on several evaluations: regular wellness council meetings, Alliance for a Healthier Generation’s School Health Index Assessment evaluations (https://schools.healthiergeneration.org/dashboard/about_assessment/, www.cdc.gov/healthyschools/shi/index.htm), and HealthCorps Community Needs Assessment evaluation at the beginning of the school year. Total exposure hours for students voluntarily participating in the program could be as long as 45 h over a maximum 36 weeks for the school year. However, the individual student-level program exposure hours varied based on attendance. Nevertheless, individual-level HealthCorps class or activities attendance records were not possible for coordinators to collect, and thus not available for the present analysis.

### Self-reported weight and height, and validation

Self-reported weight and height were collected and converted to sex-age-specific BMI z-scores following the 2000 CDC growth curves, [23] which is relevant for measuring longitudinal pediatric adiposity changes. [24] Classifications of weight status were also made based on sex-age-specific BMI percentiles: normal weight (BMI < 85th%-tile), overweight/obese (BMI ≥ 85th%-tile), and obese (BMI ≥ 95th%-tile). [25] Since only ages in years, indicated as *y* below, instead of months was available in the survey, we converted ages in years to ages in months by using the formula 12*y + 6 following the CDC guideline (www.cdc.gov/nccdphp/dnpao/growthcharts/resources/sas.htm). The self-reported weight and height were well validated by measured weight and height from an internal validation random sample of *N* = 73 students with wide ranges of height and weight: correlations between self-reported and measured weight and height were 0.89 (*p* < 0.001) and 0.91 (*p* < 0.001), respectively.

### HealthCorps survey

A 77-item HealthCorps survey for the 2013-14 academic year included questions on demographic factors, weight-related knowledge and behaviors. Survey data were collected prior to and post teaching of the HealthCorps curriculum. The survey items were selected from well-established questionnaire items such as the biannual Youth Risk Behavior Survey (YRBS) conducted by the CDC, reliabilities of whose items are well supported. [26] Reliabilities of HealthCorps survey items were assessed in our prior study [21].

Knowledge items were multiple-choice questions and had a single correct answer (see Additional file 1). Each knowledge item score was 1 if its answer was correct or 0 otherwise; when an answer was missing, we considered it incorrect. Increase of one score represents one more correct answer. The following domains of knowledge items were structured by the design of the survey questionnaires: nutrition (score range: 0-6), physical activities (score range: 0-5), breakfast (score range: 0-2), sleep (score range: 0-2), and mental resilience (score range: 0-3).

Behavior items included the same five domains as the knowledge items above. With exception of the breakfast and sleep behavior domains, the other three behavior domains included subscales representing distinctive constructs. Although the subscales of behavior items were also structured by design, determination of their final subscales was aided by factor analysis and construct validity analysis. Behavior item scores were assigned on ordinal Likert scales with higher scores representing higher frequency, higher confidence or higher likelihood. Detailed scoring schemes for each behavior item are listed in the Additional file 1. Behavior subscale scores were determined as the sum of the corresponding items.

When some, but not all, item responses were missing, the sum was prorated based on the number of observed items and their sums. The subscales of nutrition behavior domains included: Fruit and Vegetable (F&V) intake, High Energy Density (HED) food intake, Water and Juice (W&J) consumption, and Sugar-Sweetened Beverage (SSB) consumption. The subscales of physical activity domains were: Physical Activity (PA) Days, and Physical Activity (PA) Barriers. Finally, the subscales of mental resilience domains were: General Attitude, Confidence in Healthy Eating, Confidence in Exercising, and Future Exercise Plan.

### Participants

**Fig. 1:**
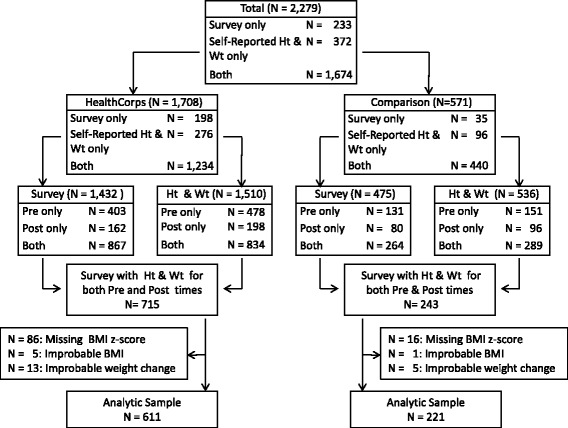
Participant flow chart

A vast majority of the students who participated in the HealthCorps program received it as a part of their school classroom programming. Therefore, their participation was not necessarily driven only by motivations. The pre-program survey was administered during September 2013 whereas the post-program survey was administered during December 2013 for the semester-long program and during May 2014 for the year-long program. A total of 2279 students (58% males) responded to either or both the pre and post survey. Among them, 1708 of the students were enrolled in classes teaching the HealthCorps curriculum, and the remaining 571 students, who were not in classes that included teaching of the HealthCorps curriculum, served as comparison students. For the present analysis, we applied the following inclusion criteria. The study sample must have: 1) responses to both surveys; 2) biologically probable BMI values [27] for both surveys; and 3) absolute difference in BMI z-scores between pre and post surveys less than 2.5 (about >3SD of sample pre-post difference) to eliminate potentially improbable weight changes. This application resulted in a total of 832 students with *N* = 611 (57.4% males) for the HealthCorps and *N* = 221 (56.6% males) for the comparison students. Detailed flowchart is depicted in Fig. 1.

### Statistical analysis

Baseline characteristics were summarized with descriptive statistics such as mean, standard deviations, and percentages. Comparisons of the characteristics between HealthCorps and comparison students at baseline were made using t- or Chi-square tests. To test our first study aim, we applied mixed-effects linear models to examine the pre-post effect of HealthCorps programming on BMI z-score to takes into account potential outcome correlations between pre and post periods in addition to clustering effects of schools; specifically, school- and participant-specific intercepts were considered random.

The main predictor for primary analysis testing significance of HealthCorps effect on changes in BMI z-sores was the time effect (pre vs. post) within each arm. To this end, we included arm indicator (HealthCorps vs. comparison), time indicator, and arm-by-time interaction as fixed effects. The arm-by-time interaction term was included to construct and test contrasts pertinent to the arm-specific time effects using the sex-specific entire sample. Likewise, for the analysis of changes in obesity rates, we applied mixed-effects logistic models with the same fixed and random effects. The same modeling approach was applied to testing the secondary aim to examine the effect of HealthCorps on knowledge and health behaviors. All of primary and secondary analyses included time, age and Hispanic ethnicity as additional fixed-effects covariates, and were conducted separately for males and females and further stratified by baseline weight status. SAS v9.3 was used for all analyses and results with *p* < 0.05 were declared statistically significant.

## Results

### Baseline characteristics (Table 1)

Distributions of age, Hispanic ethnicity, and various weight parameters were not significantly different (*p* > 0.05) between HealthCorps and comparison students regardless of sex. Sex distributions were neither significantly different between arms (*p* > 0.05).

**Table 1:** Comparisons of baseline characteristics stratified by sex

Characteristics	Males (*N* = 476): Mean (SD), %			Females (*N* = 356): Mean (SD), %		
	HealthCorps (*N* = 351)	Comparison (*N* = 125)	p	HealthCorps (*N* = 260)	Comparison (*N* = 96)	p
Age in years	15.3 (1.3)	15.4 (1.2)	0.599	15.5 (1.4)	15.4 (1.1)	0.810
Height in m	1.72 (0.09)	1.72 (0.08)	0.866	1.61 (0.08)	1.61 (0.07)	0.555
Weight in kg	66.9 (15.0)	67.7 (15.3)	0.626	60.5 (13.9)	58.4 (14.1)	0.201
BMI	22.5 (4.3)	22.8 (4.6)	0.603	23.3 (5.0)	22.6 (5.2)	0.254
BMI%-tile	60.6 (31.2)	61.4 (30.5)	0.819	64.1 (28.3)	57.9 (30.1)	0.074
BMIZ	0.35 (1.20)	0.38 (1.21)	0.781	0.47 (1.06)	0.29 (1.07)	0.140
Hispanic Ethnicity^a^	44.6%	39.5%	0.324	44.3%	37.5%	0.250
Overweight/Obese (BMI ≥ 85th%-tile)	31.1%	27.2%	0.420	31.9%	29.2%	0.618
Obese (BMI ≥ 95th%-tile)	14.3%	14.4%	0.966	12.7%	12.5%	0.961

^a^*N* = 7 and *N* = 5 are missing for males and females, respectively

### Effect of HealthCorps program on BMI z-scores (Table 2)

There was a significant decrease in the BMI z-scores for overweight/obese (BMI ≥ 85th%-tile) and obese (BMI ≥ 95th%-tile) HealthCorps female students: post-pre delta = −0.16±0.05 (*p* = 0.004) and −0.23±0.10 (*p* = 0.028), respectively. In contrast, the comparison counterparts did have a significant change in BMI z-scores: post-pre delta = −0.03±0.09 (*p* = 0.707) for overweight/obese and −0.04±0.17 (*p* = 0.825) for obese comparison female students. However, both HealthCorps and comparison normal weight male students significantly increased BMI z-scores by 0.13±0.04 (*p* = 0.001) and 0.20±0.06 (*p* = 0.002), respectively. Nevertheless, their post BMI z-scores were much lower than those of their overweight/obese and obese counterparts. We note that there were no significant changes in percentages of overweight/obese or obese students in any arm regardless of sex. These non-significant changes may be a product of the outcome scale change from the continuous BMI z-score to dichotomous obesity status.

**Table 2:** Effect of HealthCorps on BMI z-score, and overweight/obesity and obesity prevalence stratified by sex and baseline weight status

**BMI z-score**											
		HealthCorps (Est±SE)					Comparison(Est±SE)				
Sex	Baseline Weight Status	N	Pre	Post	Post–Pre delta	p	N	Pre	Post	Post–Pre delta	p
Males	Normal	242	−0.23±0.08	−0.09±0.08	**0.13±0.04**	**0.001**	91	−0.10±0.11	0.10±0.11	**0.20±0.06**	**0.002**
	Ow/Ob	109	1.68±0.08	1.61±0.08	−0.07±0.05	0.140	34	1.75±0.11	1.70±0.11	−0.05±0.08	0.513
	Obese	50	1.99±0.06	1.94±0.06	−0.05±0.07	0.496	18	2.08±0.12	1.93±0.12	−0.15±0.11	0.191
	All	351	0.36±0.09	0.44±0.09	**0.08±0.04**	**0.013**	125	0.47±0.14	0.60±0.14	**0.14±0.05**	**0.008**
Females	Normal	177	−0.04±0.06	0.03±0.06	0.07±0.04	0.069	68	−0.21±0.10	−0.20±0.10	0.02±0.06	0.728
	Ow/Ob	83	1.60±0.07	1.44±0.07	**−0.16±0.05**	**0.004**	28	1.58±0.10	1.55±0.11	−0.03±0.09	0.707
	Obese	33	1.98±0.08	1.74±0.08	**−0.23±0.10**	**0.028**	12	1.94±0.12	1.91±0.12	−0.04±0.17	0.825
	All	260	0.49±0.12	0.50±0.12	0.01±0.03	0.753	96	0.31±0.11	0.32±0.11	0.01±0.05	0.772
**Overweight/obesity and obesity rates**											
HealthCorps							Comparison				
Sex	Weight Status	Pre%	Post%	OR of Post vs. Pre (95% CI)		p	Pre%	Post%	OR of Post vs. Pre (95% CI)		p
Males	Ow/Ob	31.1%	31.3%	1.05 (0.73, 1.53)		0.785	27.2%	31.2%	1.33 (0.71, 2.48)		0.379
	Obese	14.3%	15.7%	1.22 (0.76, 1.96)		0.400	14.4%	17.6%	1.38 (0.64, 2.97)		0.412
Females	Ow/Ob	31.9%	27.7%	0.77 (0.49, 1.21)		0.251	29.2%	28.1%	0.92 (0.44, 1.96)		0.817
	Obese	12.7%	13.1%	1.08 (0.59, 19.7)		0.792	12.5%	10.4%	0.75 (0.26, 2.13)		0.583

Note: Model-based Est (Estimates) and OR (odds-ratio) were adjusted for age and Hispanic race; *SE* Standard error; *Ow/Ob* Overweight/Obese; Bold-faced numbers and *p*-values indicate significant changes from pre to post-HealthCorps teaching

### Effect of HealthCorps program on knowledge (Table 3)

Both HealthCorps male and female HealthCorps students had significant increase for all knowledge domains, expect breakfast knowledge for males. The overall knowledge score increased approximately by 1 point for both HealthCorps males and females regardless of weight status. Thus, the HealthCorps students had one more correct answer compared to pre HealthCorps program education. In contrast, no significant increases in any of knowledge domains were observed among the comparison students, males or females, except that normal weight females had a significant decrease in nutrition knowledge.

**Table 3:** Effect of HealthCorps on knowledge domains stratified by sex and baseline weight status

	Post – Pre (Estimated delta ± SE)							
	HealthCorps				Comparison			
				**Males**				
Knowledge Domains	Normal (*N* = 242)	Overweight/Obese (*N* = 109)	Obese (*N* = 50)	All (N = 351)	Normal (*N* = 91)	Overweight/Obese (*N* = 34)	Obese (*N* = 18)	All (*N* = 125)
Nutrition	**0.36±0.10**	**0.42±0.16**	0.46±0.24	**0.38±0.08**	−0.03±0.16	−0.26±0.28	−0.16±0.39	−0.10±0.14
Physical Activity	**0.29±0.09**	0.12±0.12	0.16±0.18	**0.24±0.07**	0.02±0.14	−0.21±0.21	−0.49±0.29	−0.04±0.12
Breakfast	**0.11±0.06**	0.03±0.09	0.09±0.13	0.09±0.05	0.05±0.09	0.06±0.16	0.16±0.22	0.06±0.08
Sleep	0.10±0.05	**0.20±0.08**	0.14±0.12	**0.13±0.04**	−0.04±0.09	0.06±0.14	0.06±0.20	−0.01±0.11
Mental Resilience	**0.21±0.07**	0.20±0.10	0.27±0.17	**0.20±0.06**	−0.02±0.11	−0.09±0.18	0.06±0.28	−0.04±0.09
Overall	**1.07±0.02**	**0.96±0.31**	**1.11±0.49**	**1.03±0.17**	−0.01±0.33	−0.44±0.56	−0.37±0.81	−0.15±0.29
**Females**								
	Normal (*N* = 177)	Overweight/Obese (*N* = 83)	Obese (*N* = 33)	All (N = 260)	Normal (*N* = 68)	Overweight/Obese (*N* = 28)	Obese (N = 12)	All (N = 96)
Nutrition	**0.41±0.13**	0.30±0.17	0.21±0.64	**0.38±0.10**	**−0.43±0.20**	0.31±0.27	0.32±0.39	−0.21±0.16
Physical Activity	**0.21±0.10**	0.32±0.16	0.47±0.27	**0.25±0.08**	−0.13±0.15	0.28±0.28	0.25±0.43	−0.00±0.13
Breakfast	**0.18±0.06**	0.06±0.10	0.19±0.16	**0.14±0.05**	0.10±0.10	−0.20±0.17	−0.24±0.27	0.01±0.09
Sleep	**0.24±0.06**	0.05±0.09	0.22±0.16	**0.18±0.05**	−0.03±0.09	0.07±0.16	0.33±0.26	−0.00±0.09
Mental Resilience	**0.22±0.08**	0.19±0.13	**0.49±0.20**	**0.21±0.07**	0.07±0.13	−0.03±0.21	−0.18±0.33	0.04±0.11
Overall	**1.22±0.25**	**0.90±0.36**	**1.57±0.51**	**1.13±0.21**	−0.43±0.39	0.41±0.60	0.47±0.83	−0.12±0.33

Note: Model-based Est (Estimates) were adjusted for age and Hispanic race; *SE* Standard error; Bold-faced numbers indicate significant changes from pre to post-HealthCorps teaching

### Effect of HealthCorps program on behavior (Table 4)

There was a significant increase for F&V intake and physical activities among normal weight and all male HealthCorps students. Participation in the HealthCorps program was also associated with a significant increase in confidence for exercise among obese and all male HealthCorps students. F&V intake and physical activity were also significantly increased among normal weight and all female HealthCorps students in addition to future exercise plan; however, sleep hours were significantly decreased among overweight/obese, obese and all female HealthCorps students. In contrast, obese male comparison students significantly decreased breakfast intake and sleep hours, and normal weight female comparison students significantly increased confidence in healthy eating.

**Table 4:** Effect of HealthCorps on behavior domains stratified by sex and baseline weight status

	Post – Pre (Estimated delta ± SE)							
	HealthCorps				Comparison			
**Males**								
Behavior Domains	Normal (*N* = 242)	Overweight/Obese (*N* = 109)	Obese (*N* = 50)	All (*N* = 351)	Normal (*N* = 91)	Overweight/Obese (*N* = 34)	Obese (*N* = 18)	All (*N* = 125)
Nutrition								
F&V Intake	**0.51±0.22**	0.11±0.30	0.62±0.46	**0.38±0.18**	−0.13±0.35	0.99±0.53	0.81±0.74	0.16±0.29
HED food intake	−0.19±0.32	0.39±0.40	0.35±0.62	0.00±0.25	−0.32±0.51	0.50±0.70	0.51±1.02	−0.11±0.41
W&J Intake	−0.06±0.15	−0.09±0.20	0.19±0.27	−0.07±0.12	0.08±0.25	−0.64±0.35	−0.66±0.44	−0.12±0.21
SSB Consumption	−0.07±0.18	−0.46±0.24	−0.41±0.35	−0.19±0.15	−0.06±0.29	0.75±0.42	0.89±0.56	0.16±0.24
Breakfast Intake	0.05±0.16	0.27±0.25	0.33±0.38	0.12±0.13	−0.16±0.26	−0.84±0.43	**−1.85±0.61**	−0.34±0.22
Sleep Days with >8 h	0.08±0.16	0.07±0.22	−0.14±0.36	0.08±0.013	0.26±0.26	−0.62±0.40	**−1.34±0.60**	0.04±0.22
Physical Activity								
PA Days with >1 h PA	**0.56±0.17**	0.33±0.24	0.42±0.38	**0.49±0.14**	0.22±0.28	−0.13±0.43	−0.33±0.63	0.11±0.23
PA barrier	−0.22±0.58	−0.37±0.81	−1.23±1.28	−0.27±0.48	**−1.90±0.10**	−0.21±1.42	2.74±2.08	−1.39±0.79
Mental Resilience								
General Attitude	0.05±0.08	0.10±0.13	0.11±0.22	0.07±0.07	−0.03±0.14	0.09±0.22	0.05±0.37	−0.00±0.12
Confidence in Healthy Eating	0.19±0.18	0.36±0.21	0.30±0.32	0.24±0.14	0.13±0.29	0.17±0.37	−0.67±0.54	0.14±0.23
Confidence in Exercise	0.23±0.19	0.43±0.23	**0.74±0.36**	**0.30±0.15**	0.13±0.32	0.01±0.42	0.10±0.60	0.09±0.25
Future Exercise Plan	0.52±0.37	−0.26±0.56	−0.97±0.83	0.26±0.31	−0.20±0.61	0.28±0.99	−1.64±1.39	−0.08±0.52
**Females**								
	Normal (*N* = 177)	Overweight/Obese (*N* = 83)	Obese (*N* = 33)	All (*N* = 260)	Normal (*N* = 68)	Overweight/Obese (*N* = 28)	Obese (*N* = 12)	All (*N* = 96)
Nutrition								
F&V Intake	**0.59±0.25**	0.67±0.36	0.57±0.55	**0.63±0.20**	0.35±0.40	−0.53±0.61	−0.78±0.89	0.10±0.33
HED food intake	−0.21±0.28	−0.17±0.46	0.45±0.68	−0.18±0.24	−0.00±0.44	0.54±0.79	0.15±1.14	0.16±0.39
W&J Intake	−0.22±0.17	0.18±0.21	0.33±0.36	−0.10±0.14	−0.31±0.27	0.32±0.35	0.84±0.74	−0.13±0.22
SSB Consumption	−0.18±0.18	0.09±0.27	0.27±0.35	−0.10±0.15	−0.14±0.29	−0.15±0.45	−0.51±0.59	−0.15±0.24
Breakfast Intake	−0.05±0.16	−0.34±0.30	−0.48±0.56	−0.13±0.37	0.09±0.26	0.18±0.49	−0.69±0.90	0.12±0.23
Sleep Days with >8 h	−0.24±0.19	**−0.66±0.29**	**−1.43±0.44**	**−0.36±0.16**	0.26±0.30	−0.24±0.48	−0.39±0.75	0.11±0.25
Physical Activity								
PA Days with >1 h PA	**0.66±0.18**	0.57±0.31	0.44±0.50	**0.65±0.16**	0.29±0.29	0.73±0.50	0.25±0.81	0.42±0.25
PA barrier	−0.68±0.58	0.38±1.02	0.59±1.59	−0.36±0.51	−1.08±0.94	2.11±1.67	4.69±2.58	−0.14±0.83
Mental Resilience								
General Attitude	0.16±0.10	−0.09±0.15	−0.09±0.26	0.09±0.08	0.28±0.16	0.18±0.24	0.51±0.38	0.25±0.13
Confidence in Healthy Eating	−0.04±0.17	−0.26±0.25	−0.21±0.42	−0.09±0.14	**0.62±0.27**	−0.00±0.41	−0.08±0.63	0.45±0.23
Confidence in Exercise	0.25±0.21	0.21±0.32	−0.36±0.55	0.24±0.18	0.40±0.34	0.84±0.53	0.07±0.83	0.52±0.29
Future Exercise Plan	**0.92±0.40**	0.44±0.70	0.78±1.05	**0.78±0.35**	0.79±0.63	−0.63±1.13	−1.63±1.48	0.30±0.56

Note: Model-based Est (Estimates) were adjusted for age and Hispanic race; *SE* Standard error; Bold- faced numbers indicate significant changes from pre to post-HealthCorps teaching; *F&V* Fruits & Vegetables; *HED* High-Energy Density; *W&J* Water & Juice; *SSB* Sugar-Sweetened Beverage; *PA* Physical Activity

## Discussion

The overall pattern of findings was that HealthCorps program participation resulted in BMI z-score improvement among overweight/obese and obese female students. Participation also resulted in increases in almost all knowledge domains regardless of sex in general and improved a few behavior domains. However, it did not improve weight status among male students. These findings partially support our hypothesis that the HealthCorps program would improve the weight outcome. The exact reasons for that sex difference are unclear, although similar findings in regard to sex-specific effect on weight improvements were also reported in the *Planet Health* study, a 2 year-long school-based obesity prevention study among students in grades 6 to 8. [28] Their study used school teachers for implementing their intervention, and found significant improvements only among girls. In this regard, there is precedence for our main finding. The sex differences in weight changes were not able to be explained by our data, but in general, patterns of growth in adolescence may vary by sex, and so may social pressure, readiness to change, and the extent of receptiveness to program contents.

The weight improvement among overweight or obese female student is noteworthy. The HealthCorps program did not directly intervene on weight issues per se. Rather, it engaged students in comprehensive educational and participatory activities that were designed to be age-appropriate and engaging (e.g., taste testing). Furthermore, as noted earlier, the HealthCorps school environments are in general inadequate for promoting physical activities and health behaviors. Findings concerning the effect of HealthCorps on the knowledge and behaviors are consistent with our earlier report based on a different sample of students, [21] Specifically, for both our prior and present studies, almost all knowledge domains and overall knowledge levels increased among HealthCorps students, as did F&V intakes. The significant increased knowledge levels, overall or in all domains, albeit somewhat small, were to some extent a byproduct of the program since the program focused on promoting healthier dietary choices and increasing physical activities rather than solely on increasing knowledge.

In the present study, the level of physical activity and a few mental resilience items such as confidence in exercise and future exercise plan increased significantly as well. Therefore, the present study results support our earlier findings and the robustness of HealthCorps programming pertaining to those domains included as the Dietary Guidelines 2015-2020 recommendations, [29] which emphasize physical activities and healthy dietary patterns. The construct of mental resilience is an interesting one that is understudied in the context of obesity prevention. It aligns with the construct of “GRIT”, which has been shown to predict student academic and educational outcomes. [30] This may be relevant for health and obesity-prevention behaviors for adolescents, especially those in high-risk environments, and warrants additional research.

Although overweigh/obese or obese male students tended to have reduced BMI z-score, the absence of a significant intervention effect may have been due to an insufficient dose and/or duration of the HealthCorps curriculum. Or, perhaps the attained knowledge and behavior changes for males might not have been strong enough to counteract environmental pressures outside of the school that undermine healthy weight control. Nevertheless, for males and females, future implementation strategies might need to better take into account contextual factors beyond the school that moderate treatment effects (e.g., peers and family, home and community). It might include walkability on streets or neighbors, and utilizations of public sports parks or community gardens. [31] At the individual level, setting lifestyle SMART (specific, measurable, attainable, reasonable, and time-specific) goals to improved health behaviors and reduced obesity may need to be emphasized. [32, 33]

Our study findings collectively imply that utilization of school-based comprehensive participatory educational program focusing on health knowledge and behaviors would be successful to improve students’ weight, even if such program is open to any student to ensure that it does not stigmatize students with obesity. In this context, HealthCorps program might be suitable for meeting a federal law that requires establishment of a school environment that promotes students’ health, well-being, and ability to learn by supporting healthy eating and physical activity. The Local School Wellness Policy requirement was established by the Child Nutrition and WIC Reauthorization Act of 2004, and further strengthened by the Healthy, Hunger-Free Kids Act of 2010. This legislation requires schools participating in the National School Lunch Program and/or School Breakfast Program to develop a local school wellness policy that promotes the health of students and addresses the growing problem of childhood obesity (www.fns.usda.gov/school-meals/local-school-wellness-policy).

### Limitations

The present study has several limitations that should be taken into consideration when interpreting the results and implications thereof. First, the experimental design was quasi-experimental but not a randomized design; however, that was more relevant to the real-world circumstances and settings, and the groups were comparable at baseline. Second, the analysis relied on self-reported data including weight and height. It was impossible to measure in a timely manner weights and heights of all participating students during school days not only due to logistical reasons but also due to limitation of access to students for measurements. Nevertheless, self-reported weight and height were validated by our internal sample and also supported as reliable proxy measures in other adolescent population. [34] The calculation of BMI z-scores was not based on exact ages in months but on a rough approximation of years to months as mentioned earlier. Third, individual-level potential confounding factors such as home environment and other extra-curricular physical, social or academic activities were not able to be captured. Finally, although it was necessary to enhance data quality assurance, the application of the aforementioned inclusion criteria reduced the sample size. The inclusion rate was comparable in terms of arm, sex, overweight or obesity rates, and BMI z-scores, but it was lower for older adolescents and Hispanic ethnicity. Therefore, the generalizability or transportability of findings to these adolescent populations may be limited.

## Conclusions

In conclusion, despite the fact that the HealthCorps school settings were challenging especially in terms of adequate resources, it was effective for BMI z-score improvements among female students in addition to significant positive effects on knowledge and a few behaviors in both sexes. It remains an open question as to how one can develop implementation strategies concerning school-based extra or intramural program that takes into account home and community contextual factors at the societal level and SMART goals approach at the individual level to improve health behaviors, further weight improvement, and reduce adolescent obesity prevalence. To this end, future studies need to address how to design and evaluate strategies for achieving and sustaining adolescent weight improvement while meeting academic and other challenges in urban inner city high schools.

## Additional file

Additional file 1: Knowledge and Behavior Domains and Subscales**.** HealthCorps 2013-2014 survey items and scoring schemes for knowledge, nutrition, physical activity and mental health domains and subscales. (DOCX 28 kb)

## Abbreviations

BMI: Body Mass Index
CDC: Centers for Diseases Control and Prevention
F&V: Fruits & Vegetables
HED: High-Energy Density
NYC: New York City
PA: Physical Activity
SMART: Specific, Measurable, Attainable, Reasonable, and Time-specific
SSB: Sugar-Sweetened Beverage
W&J: Water & Juice
WIC: Women, Infants and Children
YRBS: Youth Risk Behavior Survey
