# Correction to: Effective nationwide school-based participatory extramural program on adolescent body mass index, health knowledge and behaviors

**DOI:** 10.1186/s12887-018-1053-7

**Published:** 2018-02-26

**Authors:** Moonseong Heo, Camille C. Jimenez, Jean Lim, Carmen R. Isasi, Arthur E. Blank, David W. Lounsbury, Lynn Fredericks, Michelle Bouchard, Myles S. Faith, Judith Wylie-Rosett

**Affiliations:** 1 Department of Epidemiology and Population Health, Albert Einstein College of Medicine, 1300 Morris Park Avenue, Bronx, NY 10461 USA; 2 HealthCorps, 33 Irving Pl, 3rd Floor, New York, NY 10003 USA; 3 Department of Family and Social Medicine, Department of Epidemiology and Population Health, Albert Einstein College of Medicine, Harold and Muriel Block Building, Room 409, 1300 Morris Park Avenue, Belfer 13-th floor, Bronx, NY 10461 USA; 4 FamilyCook Productions, 330 East 43rd street, Ste. 704, New York, NY 10017 USA; 5 Department of Counseling, School, and Educational Psychology, Graduate School of Education, University of Buffalo – SUNY, 420 Baldy Hall, Buffalo, NY 14260-1000 USA

## Correction

Following the publication of the original article [1], it was brought to our attention that author Judith Wylie-Rosett was erroneously included as Judith Wylie.

The full author’s name and surname is included in the author list of this ‘Correction’ and has been updated in the original article [1].
