# Supplementary material for: Effective nationwide school-based participatory extramural program on adolescent body mass index, health knowledge and behaviors
Source: BMC Pediatr. 2018 Jan 16;18:7. doi: 10.1186/s12887-017-0975-9 (PMC5771213; doi:10.1186/s12887-017-0975-9)
Supplement: Additional file 1: — Knowledge and Behavior Domains and Subscales. HealthCorps 2013-2014 survey items and scoring schemes for knowledge, nutrition, physical activity and mental health domains and subscales. (DOCX 28 kb) [file 12887_2017_975_MOESM1_ESM.docx]

**SUPPLEMENATRY MATERIAL**: Knowledge and Behavior Domains and Subscales

A, Knowledge Domains and Score Ranges

1. Nutrition Knowledge Domain Questions: (Score Range: 0-6)

- The three macronutrients that your body needs to survive are:
- Food items that are NOT good sources of protein:
- The best kind of carbohydrates are:
- This is NOT a purpose of eating fats:
- The formula to determine the total amount of calories in a soda is:
- One-half of the foods on your plate should be from:

2. Physical Activity knowledge Domain Questions (Score Range: 0-5)

- Regular exercise can make you feel happy.
- Regular exercise can make you grow taller.
- Regular exercise can make you concentrate better.
- Exercises like crunches or planks work this body region:
- This is a TRUE statement about exercise:

3. Breakfast Knowledge Domain Questions: (Score Range: 0-2)

- The nutrients included in a healthy breakfast are:
- This is NOT true about high school students who eat breakfast:

4. Sleep Knowledge Domain Questions: (Score Range: 0-2)

- This is a TRUE statement about technology (cell phones, computers, television, etc.) and our sleep:
- This is a consequence of too little sleep for teenagers:

5. Mental Health Knowledge Domain Questions (Score Range: 0-3)

- This is an example of optimism:
- This is NOT a positive way to deal with a situation that makes you angry:
- This is NOT a positive method for stress reduction:

B. Behavior Domains, Subscales and Score Ranges

1. Nutrition Behavior Domain Subscales:

1.1 Fruit & Vegetables intake (F&V; Score Range: 4-18): *Think about* last month *and indicate how often you ate or drank each of the following:*

|  | Score | | | | | |
| --- | --- | --- | --- | --- | --- | --- |
|  | Many times a day | Once a day | Once every couple of days | Once a week | A few times in the month | Never |
| Item | 6 | 5 | 4 | 3 | 2 | 1 |
| Fresh fruit | (A) | (B) | (C) | (D) | (E) | (F) |
| Raw or cooked vegetables | (A) | (B) | (C) | (D) | (E) | (F) |
| Green salad | (A) | (B) | (C) | (D) | (E) | (F) |

1.2 High Energy Density food intake (HED: score range: 4-24)*Think about* last month *and indicate how often you ate or drank each of the following:*

|  | Score | | | | | |
| --- | --- | --- | --- | --- | --- | --- |
|  | Many times a day | Once a day | Once every couple of days | Once a week | A few times in the month | Never |
| Item | 6 | 5 | 4 | 3 | 2 | 1 |
| Hamburger, hot dog, or sausage | (A) | (B) | (C) | (D) | (E) | (F) |
| Grilled or roasted chicken | (A) | (B) | (C) | (D) | (E) | (F) |
| French fries or potato chips | (A) | (B) | (C) | (D) | (E) | (F) |
| Cookies, doughnuts, cake, or candy | (A) | (B) | (C) | (D) | (E) | (F) |

1.3 Water and Juice consumption (W&J; score range: 4-24): *Think about* last month *and indicate how often you ate or drank each of the following:*

|  | Score | | | | | |
| --- | --- | --- | --- | --- | --- | --- |
|  | Many times a day | Once a day | Once every couple of days | Once a week | A few times in the month | Never |
| Item | 6 | 5 | 4 | 3 | 2 | 1 |
| Water | (A) | (B) | (C) | (D) | (E) | (F) |
| 100% fruit juice such as orange juice or apple juice | (A) | (B) | (C) | (D) | (E) | (F) |

1.4 Sugar-Sweetened Beverage consumption (SSB; score range: 2-12): *Think about* last month *and indicate how often you ate or drank each of the following:*

|  | Score | | | | | |
| --- | --- | --- | --- | --- | --- | --- |
|  | Many times a day | Once a day | Once every couple of days | Once a week | A few times in the month | Never |
| Item | 6 | 5 | 4 | 3 | 2 | 1 |
| Fruit-flavored drinks such as Arizona Iced Tea, Gatorade, or fruit punch | (A) | (B) | (C) | (D) | (E) | (F) |
| Can, bottle, or glass of regular soda such as Coke, Pepsi, or Sprite | (A) | (B) | (C) | (D) | (E) | (F) |

2. Breakfast Behavior Domain

Breakfast intake (Score Range: 1-8): During the PAST SEVEN DAYS, indicate the number of days you ate breakfast:

| Response | Score | Response | Score |
| --- | --- | --- | --- |
| (A) None | 1 | (E) 4 days | 5 |
| (B) 1 day | 2 | (F) 5 days | 6 |
| (C) 2 days | 3 | (G) 6 days | 7 |
| (D) 3 days | 4 | (H) 7 days | 8 |

3. Sleep Behavior Domain:

Sleep days (Score Range: 1-8): During the PAST SEVEN DAYS, indicate the number of days you got at least 8 hours of sleep:

| Response | Score | Response | Score |
| --- | --- | --- | --- |
| (A) None | 1 | (E) 4 days | 5 |
| (B) 1 day | 2 | (F) 5 days | 6 |
| (C) 2 days | 3 | (G) 6 days | 7 |
| (D) 3 days | 4 | (H) 7 days | 8 |

4. Physical Activity Behavior Domain Subscales:

4.1 Physical Activity Days (PA Days; score range: 1-8): During the PAST SEVEN DAYS, the number of days you did at least 1 hour of exercise or physical activity:

| Response | Score | Response | Score |
| --- | --- | --- | --- |
| (A) None | 1 | (E) 4 days | 5 |
| (B) 1 day | 2 | (F) 5 days | 6 |
| (C) 2 days | 3 | (G) 6 days | 7 |
| (D) 3 days | 4 | (H) 7 days | 8 |

4.2 Physical Activity Barriers (PA Barriers; score range: 5-50): Indicate about how often these things PREVENT you from getting exercise

|  | Score | | | | |
| --- | --- | --- | --- | --- | --- |
|  | Very Often | Often | Sometimes | Rarely | Never |
| Item | 5 | 4 | 3 | 2 | 1 |
| Self-conscious about your looks when you exercise or do physical activities | (A) | (B) | (C) | (D) | (E) |
| Lack of self-discipline | (A) | (B) | (C) | (D) | (E) |
| Lack of time | (A) | (B) | (C) | (D) | (E) |
| Lack of energy/too tired to exercise | (A) | (B) | (C) | (D) | (E) |
| Don’t have anyone to do the exercise/physical activities with me | (A) | (B) | (C) | (D) | (E) |
| Don’t enjoy exercising or doing physical activities | (A) | (B) | (C) | (D) | (E) |
| Lack of skills or knowledge about how to exercise | (A) | (B) | (C) | (D) | (E) |
| Lack of convenient place to exercise | (A) | (B) | (C) | (D) | (E) |
| Belief you are too overweight | (A) | (B) | (C) | (D) | (E) |
| Don’t like to sweat/mess up your appearance (hair, clothes, make-up) | (A) | (B) | (C) | (D) | (E) |

5. Mental Resilience Domain Subscales:

5.1 General Attitude (score range: 4-8): Indicate the ONE sentence in each pair that is more like YOU:

| Score |  |  |
| --- | --- | --- |
| 2 |  | 1 |
| (A) I am generally happy with my appearance | *OR* | (B) I am very concerned about my appearance |
| (A) I am often happy | *OR* | (B) I am usually not happy |
| (A) I usually like the kind of person I am | *OR* | (B) I often wish I was someone else |
| (A) I tend to think positively much of the time | *OR* | (B) I tend to think negatively much of the time |

5.2 Confidence in Healthy Eating (Score Range: 4-12): Indicate the extent to which you feel confident you can achieve the following goals

|  | Score | | |
| --- | --- | --- | --- |
|  | Very confident | Somewhat confident | Not confident |
| Item | 3 | 2 | 1 |
| Eating healthy even if you have to try several times until it works | (A) | (B) | (C) |
| Eating healthy even if you have to rethink your entire way of nutrition | (A) | (B) | (C) |
| Eating healthy even if you do not receive a great deal of support from others | (A) | (B) | (C) |
| Eating healthy even if you have to make a detailed plan | (A) | (B) | (C) |

5.3 Confidence in Exercising (Score Range: 4-12): Indicate the extent to which you feel confident you can achieve the following goals

|  | Score | | |
| --- | --- | --- | --- |
|  | Very confident | Somewhat confident | Not confident |
| Item | 3 | 2 | 1 |
| Exercising even when you have worries and problems | (A) | (B) | (C) |
| Exercising even if you feel depressed | (A) | (B) | (C) |
| Exercising even when you are tired | (A) | (B) | (C) |
| Exercising even when you are busy | (A) | (B) | (C) |

5.4 Future Exercise Plan (Score Range: 3-15): Think about the future and indicate how likely you will be doing regular exercise or physical activity during each of the following time periods in your life:

|  | Score | | | | |
| --- | --- | --- | --- | --- | --- |
|  | I definitely will NOT | I probably will NOT | About a 50/50 chance | I probably WILL | I definitely WILL |
| Item | 1 | 2 | 3 | 4 | 5 |
| Next summer | (A) | (B) | (C) | (D) | (E) |
| Outside of school, one year from now | (A) | (B) | (C) | (D) | (E) |
| When you are an adult | (A) | (B) | (C) | (D) | (E) |
